# Insects in Drugs

**Published:** 1890-03-01

**Authors:** 


### INSECTS IN DRUGS.

It is reported in the *American Druggist* last received that at a meeting of the Chemists Association, New York, Mr. C. J. Strother showed a number of drugs infected with animal life. The first was a fair looking "sample of crushed linseed, which, seen under a lens, was found to be literally swarming with parasites." Mr. Strother asked the question, what would be the effect of a poultice containing thousands of insect applied to an open wound, especially if the poultice be made with hot instead of boiling water? To this we cannot reply, and presume it does not matter much, as we have recently been told (*Brit. Med. Jour.*, February 25th) that poultices are now "remedies belonging to ancient history." Other drugs containing insects were aconite root, nux vomica, and cantharides, the parasites in which were all different. It is said what is one man's meat is another man's poison. And it would appear that the same applies to insects. Cockroaches we know are very fond of red pepper, especially if presented to them in the form of a ripe pod.

